# Engineering Charge Transfer Characteristics in Hierarchical Cu_2_S QDs @ ZnO Nanoneedles with p–n Heterojunctions: Towards Highly Efficient and Recyclable Photocatalysts

**DOI:** 10.3390/nano9010016

**Published:** 2018-12-23

**Authors:** Donglai Han, Boxun Li, Shuo Yang, Xinying Wang, Wei Gao, Zhenjun Si, Qinghui Zuo, Yanhui Li, Yanwei Li, Qian Duan, Dandan Wang

**Affiliations:** 1 School of Materials Science and Engineering, Changchun University of Science and Technology, Changchun 130022, China; 18624090462@163.com (B.L.); szj@cust.edu.cn (Z.S.); zuoqinghui@cust.edu.cn (Q.Z.); liyanhui@ciac.ac.cn (Y.L.); liyanwei@cust.edu.cn (Y.L.); 2 Engineering Research Center of Optoelectronic Functional Materials, Ministry of Education, Changchun 130022, China; 3 Changchun Institute of Optics, Fine Mechanics and Physics, Chinese Academy of Sciences, Changchun 130033, China; yangshuo_2011@163.com; 4 School of Engineering and Architecture, Northeast Electric Power University, Jilin City 132012, China; wangxinying.2008@163.com; 5 School of Materials Science and Engineering, Jilin University, Changchun 130025, China; handonglaijiayou@126.com; 6 Quality and Reliability Assurance Department, GLOBALFOUNDRIES (Singapore) Pte. Ltd., 60 Woodlands Industrial Park D, Street 2, Singapore 738406, Singapore

**Keywords:** QDs, ZnO@Cu_2_S hierarchical structure, p-n heterojunctions, Photocatalysis, Photostability

## Abstract

Equipped with staggered gap p-n heterojunctions, a new paradigm of photocatalysts based on hierarchically structured nano-match-shaped heterojunctions (NMSHs) Cu_2_S quantum dots (QDs)@ZnO nanoneedles (NNs) are successfully developed via engineering the successive ionic layer adsorption and reaction (SILAR). Under UV and visible light illumination, the photocatalytic characteristics of Cu_2_S@ZnO heterojunctions with different loading amounts of Cu_2_S QDs are evaluated by the corresponding photocatalytic degradation of rhodamine B (RhB) aqueous solution. The results elaborate that the optimized samples (S3 serial specimens with six cycles of SILAR reaction) by means of tailored the band diagram exhibit appreciable improvement of photocatalytic activities among all synthesized samples, attributing to the sensitization of a proper amount of Cu_2_S QDs. Such developed architecture not only could form p–n junctions with ZnO nanoneedles to facilitate the separation of photo-generated carries but also interact with the surface defects of ZnO NNs to reduce the electron and hole recombination probability. Moreover, the existence of Cu_2_S QDs could also extend the light absorption to improve the utilization rate of sunlight. Importantly, under UV light S3 samples demonstrate the remarkably enhanced RhB degradation efficiency, which is clearly testified upon the charge transfer mechanism discussions and evaluations in the present work. Further supplementary investigations illustrate that the developed nanoscale Cu_2_S@ZnO heterostructures also possess an excellent photo-stability during our extensive recycling photocatalytic experiments, promising for a wide range of highly efficient and sustainably recyclable photocatalysts applications.

## 1. Introduction

With the increasing emphasis on environmental pollution and energy shortage, photocatalytic degradation of organic pollutants in water using solar energy has become a promising route to solve those problems due to its environmental friendliness, high efficiency, and easy operation compared to traditional treatment methods, such as coagulation, adsorption, and membrane separation [1,2,3,4]. Therefore, great deals of efforts have been devoted in recent years to improve the necessitating high efficiency, long-term stability and low cost semiconductor photocatalysts [5,6,7,8,9,10]. Among various semiconductor materials, zinc oxide (ZnO) has attracted significant attention because of its superior characteristics, e.g., direct wide band gap (*E*_g_ = 3.37 eV), easy access, low-cost, low-toxic, high photosensitivity, chemical stability, tunable nanoscale size with adjustable optical and magneto-optical, as well as perfect electron mobility (205–300 cm, 2 V/s) [11,12,13,14,15,16,17,18,19]. Nevertheless, under UV light, the fast electron-hole pair recombination and photocorrosion in the single phase ZnO leads to a low photocatalytic activity, and ZnO under visible light is almost no activity by reason of the high band gap [20,21].

To overcome the drawbacks of ZnO, we need to develop a new material with better optical absorption capacity and lower tendency towards charge recombination than that of pure ZnO material. Until now, possible solutions have been tested. Metals doping (such as gold, silver, rhodium, or platinum) [22,23], metal ions doping (such as Bi or Al) [24], and nitrogen doping [25] are the most used techniques to give rise to visible light activity of the semiconductor materials, to extend their light absorption ability from UV to visible range and to feature good electron transport. Beyond that, heterojunctions, which can effectively capture the photo-generated charge carriers to improve the charge separation efficiency, decrease the surface reaction over potential, enhance apparent quantum efficiencies and provide active sites for surface redox reaction at two different reaction sites, are believed to be essential to achieving highly efficient photocatalytic performances [26,27,28,29,30,31,32,33,34,35,36,37]. Recently, semiconductor heterojunctions composed of ZnO and other metal sulfides or oxides have also been extensively studied, for example combining ZnO with Cu_2_O, CdS, SnO_2_, MoO_3,_ and TiO_2_ semiconductor materials [1,2,32,33,38,39,40]. Aforementioned results demonstrate that the composites developed by coupling different semiconductor materials could exhibit collective and enhanced property by reciprocal transfer of electrons and holes from one semiconductor to the other under irradiation and, consequently, realizing a higher photocatalytic activity [41,42,43]. Copper sulfides (Cu_2_S), as an important p-type semiconductor due to the stoichiometric deficiency of copper in the lattice, are holding great potential applications in diverse fields including cold cathodes [44], solar cells [45], nanoscale switches [46], chemical sensing [47] and nanoscale switches [48]. In addition, attributed to its excellent combination of a bulk band gap of 1.2 eV, an absorption coefficient of >10^4^ cm^−1^, a certain thermal and chemical stability, the elemental abundance as well as low toxicity [49], Cu_2_S has also been considered as an ideal light absorbing material for photocatalytic [12], photothermal [50], photovoltaic [51] and optoelectronic [2] applications. The band structures of Cu_2_S and ZnO are sufficient to facilitate the electron transfer process. In this process, the photo-generated electrons can flow from Cu_2_S to ZnO, and the charge carriers become physically separated once they are generated [52]. Therefore, the Cu_2_S and ZnO have been selected to compose a highly catalytic efficient, stable and cost-effective p–n heterostructure. 

Different preparation methods for fabrication ZnO semiconductor materials such as coprecipitation [53], micro-emulsions [54], sol-gel [55], hydrothermal routes [56], combustion [57], pulsed-laser deposition [58], spray pyrolysis [59], etc. have been used. And various synthesis methods for fabrication Cu_2_S semiconductor materials such as successive ion layer adsorption (SILAR) [60], physical vapor deposition (PVD) [61], hydrothermal method [62], solvothermal decomposition [63], etc. have also been tested. Of all the preparation methods, hydrothermal method for ZnO and deposition Cu_2_S on substrate by SILAR have been proved to be the simpler, cheaper, and less toxic methods [64].

In the present work, we report on the synthesis of p-n heterostructure comprising of ZnO nanoneedles (NNs) decorated with Cu_2_S quantum dots (QDs) by a low-cost, easy to operate and environmentally friendly two step method. The first step is to prepare ZnO NNs by hydrothermal method, and the second step is to deposit Cu_2_S QDs on ZnO NNs by SILAR method. The photocatalytic performances of ZnO NNs and ZnO@Cu_2_S nano-match-shaped heterojunctions (NMSHs) under UV and visible light irradiation were evaluated by the photocatalytic degradation of rhodamine B (RhB). The structural characteristics of ZnO@Cu_2_S NMSHs and the role of Cu_2_S QDs in improving the photocatalytic activity of ZnO NNs are the focus of the present study and discussions.

## 2. Materials and Methods

### 2.1. Materials

Ethylenediamine, Copper nitrate trihydrate (Cu(NO_3_)_2_·3H_2_O), sodium sulfide nonahydrate (Na_2_S·9H_2_O), RhB and other required chemicals were all analytical-grade reagents and were used as received without further purification. And all of them were obtained from Sinopharm Chemical Regent Co., Ltd. (Shanghai, China).

### 2.2. Samples Preparation

**Figure 1:**
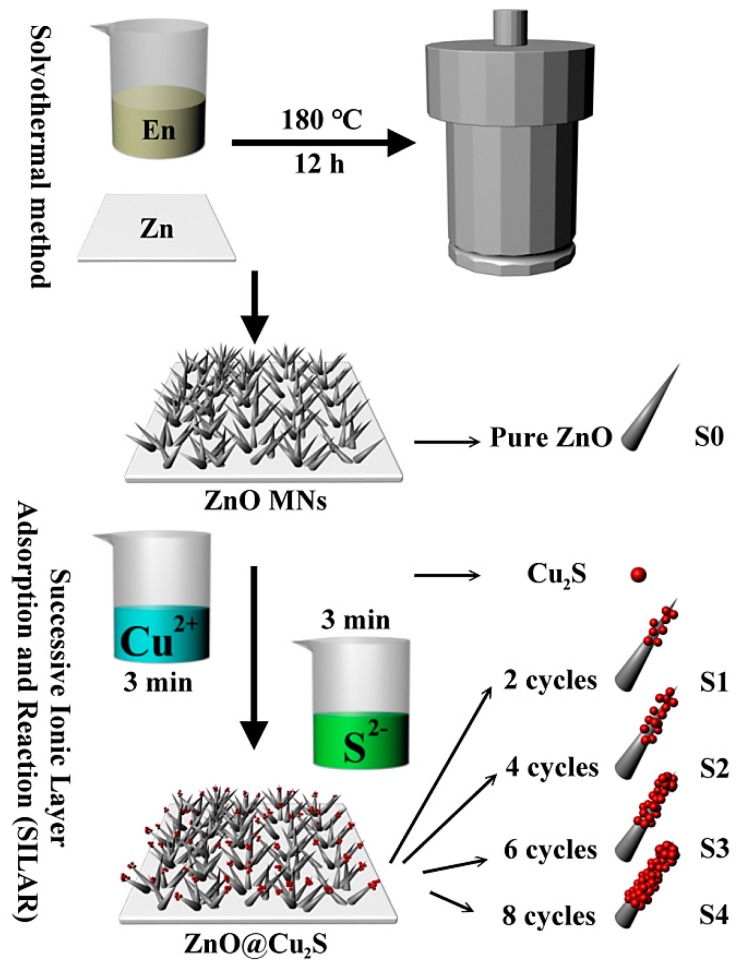
Schematic of ZnO@Cu_2_S nano-match-shaped heterojunctions (NMSHs) synthesis.

The synthesis processes of ZnO@Cu_2_S NMSHs can be divided into two steps: ZnO NNs growing and Cu_2_S coating, as shown in Figure 1.

**ZnO NNs growth:** Zinc sheet with the size of 1.0 cm × 1.0 cm (width × width), the thickness of 0.22 mm and the purity of 99% was used as zinc source and a substrate for direct growth ZnO NNs. First Zinc sheet was put into a mixed aqueous solution containing 5 mL ethylene diamine and 5 mL deionized water. Then, this system was transferred into a 20 mL Teflon-lined autoclave and kept at 180 °C for 12 h as Figure 1 shows. After the reaction, the autoclave was taken out and cooled down to room temperature. The products (each thin film) were thoroughly rinsed with deionized water and absolute ethanol. Ultimately, the pure ZnO NNs were obtained and labeled as S0.

**Cu_2_S QDs synthesis and coating:** Due to the quantum dots possess few atoms and small size effect, it can generate special interface effects and function under the quantum confinement effect [65,66,67,68]. In our experiments, ZnO@Cu_2_S NMSHs were prepared through a modified successive ionic layer adsorption and reaction (SILAR) method. Firstly, the prepared ZnO NNs were immersed into the Cu(NO_3_)_2_ (0.5 mol/L) solution and remained stationary for 3 min for adsorbing Cu^2+^ ions, and then the samples were taken out and washed thoroughly with deionized water to remove the excess Cu^2+^ ions which adsorbed weakly on the surface of samples. Secondly, we continued to place the samples in the Na_2_S (0.5 mol/L) solution and repeated the above operation. When the tip of ZnO NNs begun to turn black, it proved that Cu_2_S QDs has begun to adsorb to the ZnO NNs and the ZnO@Cu_2_S NMSHs were initially synthesized. In order to investigate the effect of the deposition amount of Cu_2_S QDs on photocatalytic performance, samples with repeating different SILAR cycle times were prepared, and the samples with two, four, six and eight SILAR cycle times were denoted as S1, S2, S3, S4, respectively. The surface of the ZnO NNs turned dark when we repeat this SILAR cycle for eight times, indicating that Cu_2_S QDs has completely covered the tip of the ZnO NNs and the ZnO@Cu_2_S NMSHs have been compounded.

### 2.3. Characterization

The X-ray diffraction patterns (XRD) of the as-prepared samples were measured on a D/max-2500 copper rotating-anode X-ray diffractometer (Rigaku Corporation, Tokyo, Japan) with Cu Kα radiation of wavelength λ = 1.5406Å (40 kV, 200 mA). The surface morphologies of the as-prepared samples were characterized by a field emission scanning electron microscope (FESEM, 7800F, JEOL Ltd., Tokyo, Japan), and the elemental composition was estimated by energy-dispersive X-ray spectroscopy (EDX) (JEOL Ltd., Tokyo, Japan). Transmission electron micrographs (TEM) and high-resolution transmission electron microscopy (HRTEM) images were taken on a FEI Tenai G2 F20 electron microscope (JEOL Ltd., Tokyo, Japan) equipped with an X-ray energy dispersive spectrometer (EDS) (JEOL Ltd., Tokyo, Japan). Chemical components and the binding energies of ZnO NNs and the ZnO@Cu_2_S NMSHs were analyzed by X-ray photoelectron spectroscopy (XPS) (Thermo Scientific ESCALAB 250Xi A1440 system, Thermo Fisher Scientific, Waltham, MA, USA). Photoluminescence (PL) spectra were investigated at room temperature on a Renishaw in Via micro-PL spectrometer (Renishaw, London, UK) at room temperature (λ_ex_ = 325 nm, He-Cd laser). The UV-Vis diffuse reflection spectra (DRS) of the samples (S0-S4) were measured by an UV-Vis spectrophotometer (UV-5800PC, Shanghai Metash Instruments Co., Ltd., Tokyo, Japan). 

### 2.4. DRS Test and Photocatalyic Test

**DRS test:** First, install the integrating sphere attachment. Then, in the measurement method of the UV Probe software, the measurement method is set to absorbance, and the measurement wavelength range is set. A standard sample is placed on the sample sit of the integrating sphere. Baseline correction was performed over the measurement wavelength range. Finally, the standard sample of the integrating sphere is removed and replaced with the sample to be measured.

**Photocatalytic test:** The photocatalytic activities of the obtained samples were measured by the degradation of RhB aqueous solution under UV and visible light irradiation. A 250 W high-pressure mercury lamp with average light intensity of 22.11 mW/cm^2^ (main wavelength 365 nm) was used as UV source and a 300 W Xe arc lamp with the intensity of 5 W·cm^−2^ were used as the visible light source by a 420nm cutoff filter. After washing with the RhB aqueous solution (2 mg/L), the square substrates (covered with ZnO NNs or ZnO@Cu_2_S NMSHs) with the size of 1.0 cm × 1.0 cm (width × width) were immersed into the RhB aqueous solution for 20 min in the dark to reach an adsorption-desorption equilibrium between the catalysts and RhB molecules. After that, the light source was switched on, and then 2mL of aliquots was withdrawn from the irradiated suspension every 20 min. The concentrations of RhB before and after different irradiation intervals were analyzed by a UV-Vis spectrophotometer with 14 cm away between the cuvettes and the light source, and then the percentage degradation was calculated. 

## 3. Results and Discussion

### 3.1. Morphologies and Phase Structures

**Figure 2:**
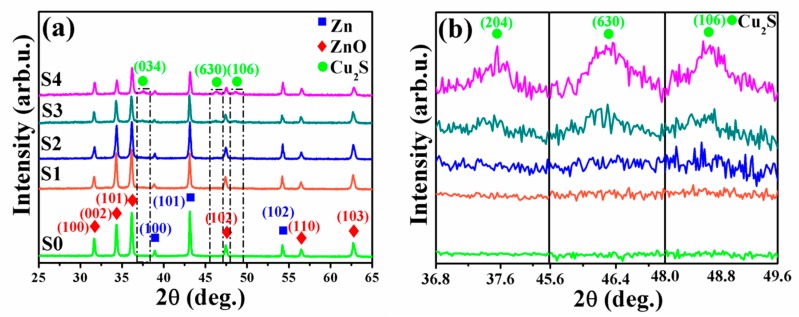
Wide-range (**a**) and magnified (**b**) XRD patterns of ZnO NNs (S0) and ZnO@Cu_2_S NMSHs with depositing Cu_2_S QDs for two (S1), four (S2), six (S3), and eight (S4) cycles by the successive ionic layer adsorption and reaction (SILAR) method.

Figure 2a,b show the XRD and partially magnified XRD patterns (the black rectangle in Figure 2a) of ZnO NNs (S0) and ZnO@Cu_2_S NMSHs (S1–S4). The pronounced diffraction peaks were exhibiting the crystalline nature, so the average crystallite size was determined using the Scherrer’s formula, *D* = 0.9 λ/β Cosθ, where λ is the wavelength of x-ray radiation, β is the full width of half maximum of the peak at diffracting angle θ. The average crystallite size of the S0–S4 samples remained virtually unchanged as the increasing amount of Cu_2_S QDs in ZnO@Cu_2_S NMSHs, which indicate that copper is not doped into the bulk phase of ZnO but exists in the form of sulfide [69]. It can be clearly seen from Figure 2a that all the diffraction peaks of as-synthesized S0, S1, and S2 samples agree well with the hexagonal wurtzite phase structure of ZnO (JCPDS card No.36-1451) [70], except for some peaks (marked with blue squares) coming from the Zn (JCPDS No. 87-0713) substrate. However, these diffraction peaks of Cu_2_S are absence in the XRD patterns of S1 and S2 samples (Figure 2b), which may be attributed to the low loading amount of Cu_2_S nanomaterials loaded on the surface of ZnO. This will be further discussed by the SEM and TEM test. As the XRD patterns of S3 and S4 samples shown, the diffraction peaks of as-synthesized ZnO@Cu_2_S NMSHs observed at 2θ values of 31.7°, 34.3°, 36.2°, 47.4°, 56.5°, and 62.7° are matched well to (100), (002), (101), (102), (110), and (103) planes of the hexagonal wurtzite ZnO (space group p63mc, JCPDS card No.36-1451) [70], meanwhile the observed at 2θ values of 37.1°, 45.6°, and 47.9° could be indexed to (204), (630), and (106) planes of chalcocite Cu_2_S phase (JCPDS card No. 73-1138). No characteristic peaks of other impurities were detected, indicating that the films were prepared as we designed.

**Figure 3:**
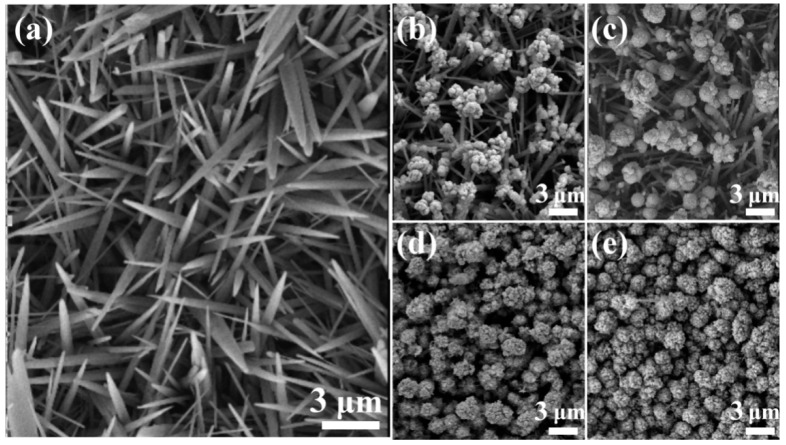
Field emission scanning electron microscope (FESEM) images of ZnO NNs and ZnO@Cu_2_S NMSHs with depositing Cu_2_S QDs for different cycles by SILAR method. (**a**) ZnO NNs (S0), (**b**) ZnO@Cu_2_S NMSHs (S1, two cycles), (**c**) ZnO@Cu_2_S NMSHs (S2, four cycles), (**d**) ZnO@Cu_2_S NMSHs (S3, six cycles), and (**e**) ZnO@Cu_2_S NMSHs (S4, eight cycles).

Field emission scanning electron microscopy (FESEM) images morphology evolution investigations were carried out to observe the amount and morphology of ZnO NNs and Cu_2_S QDs in the ZnO@Cu_2_S NMSHs. Figure 3a reveals the FESEM image of the pure ZnO NNs (S0) and Figure 3b–e reveal the FESEM images of ZnO@Cu_2_S NMSHs synthesized with SILAR method for two, four, six, and eight cycle times, respectively. As shown in Figure 3a, the ZnO nanocrystals with very clean and smooth surface exhibit needle-like structure, and the diameter and length range of the pure ZnO NNs (S0) are 340~530 nm and up to 5.3~10.6 μm, respectively. From Figure 3b–e we can see that the shapes of S1–S4 samples are like “matches” with different sizes of heads proving that the Cu_2_S QDs has been deposited on the ZnO NNs. As the loading amount of Cu_2_S QDs was improved, the average sizes of the head of these “matches” samples (S1–S4) grow to 670 nm, 1.3, 2.0, and 2.3 μm, respectively. When the SILAR reaction cycle reaches six times (S3), the surfaces of ZnO NNs were nearly covered by the Cu_2_S QDs and the nanoneedles structure of ZnO was hardly detected (Figure 3d). Meanwhile, plenty of Cu_2_S QDs became to aggregate together (Figure 3d). Particularly, when the SILAR reaction cycle reaches eight times (S4), the heads of those “matches” have been tightly connected to each other to form a Cu_2_S shell layer that closely covers ZnO nanoneedles.

**Figure 4:**
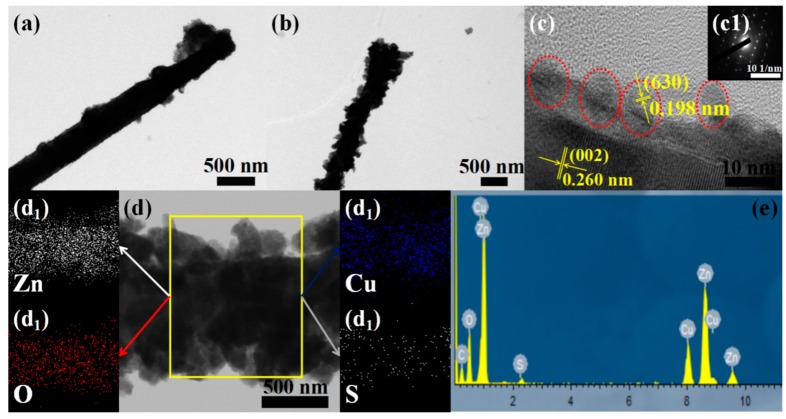
(**a**) TEM images of S1 (depositing Cu_2_S QDs for two cycles by SILAR method); (**b**–**d**) TEM and HRTEM images of S3 (depositing Cu_2_S QDs for six cycles by SILAR method); (**c1**) SAED images of S3; (**d1**–**d4**) the EDS elemental mappings for Zn, O, Cu and S of S3 in (d); (**e**) EDX spectra of S3.

In order to further identify the morphology and detailed structure of the ZnO@Cu_2_S NMSHs and reveal its chemical element composition, TEM (Figure 4a) test of S1 sample and TEM (Figure 4b,d), HRTEM (Figure 4c), selected area electron diffraction (SAED, Figure 4c1), EDX, and EDS elemental mappings for Zn, O, Cu, and S (Figure 4e and Figure 4d1–d4) analysis of S3 sample was carried out. When Cu_2_S was deposited to form ZnO@Cu_2_S NMSHs (S1 and S3 samples), it was observed in Figure 4a,b that a number of Cu_2_S QDs were selectively grown on the tips of ZnO NNs to form a match-like morphology composites, and as the number of Cu_2_S QDs deposition increases, the size of the top end of the match-shaped nanomaterial increases from 680 to 910 nm. The HRTEM images of the ZnO@Cu_2_S NMSHs (Figure 4c) and the TEM images (Figure 4d) show an apparent contrast between the inner core and the outer shell, which suggest the existence of a core-shell structure. The HRTEM image in Figure 4c clearly shows that the shell has an interlayer spacing of 0.198 nm, which is in agreement with the lattice spacing of the (630) planes of the chalcocite Cu_2_S (*d*_(630)_ = 0.197 nm for bulk Cu_2_S); the core displays an interlayer spacing of 0.26 nm, which agrees well with the lattice spacing of the (002) planes of the hexagonal wurtzite-type ZnO (*d*_(002)_ = 0.26 nm for bulk wurtzite ZnO). At the same time, we can also get the information from Figure 4c that the as-prepared Cu_2_S QDs with an average diameter of 7 nm are uniformly distributed on the surface of the ZnO NNs, and the Cu_2_S QDs and the ZnO NNs grow preferentially along the [630] and [002] direction, respectively. The corresponding SAED pattern in Figure 4c1 reveals that ZnO NNs has a single crystal-like structure and Cu_2_S QDs has a polycrystalline structure which further confirms that the sample we prepared is a composite material consisting of ZnO and Cu_2_S. Figure 4d was selected to do the EDS elemental mapping test and the Figure 4d1–d4 display the EDS elemental mapping images of Zn, O, Cu and S, respectively. As shown in Figure 4d1–d4, the distributions of Zn, O, Cu, and S atoms are uniformly dispersed and the Zn element is concentrated only at the core region while the Cu signal is dispersed in the entire nanoneedles, which again confirms the core-shell configurations with the Cu_2_S sheath have been generated. Compare Figure 4d1 with Figure 4d3, the distribution density of the S element is much lower than that of the Cu element, which indicates that the valence of Cu is lower than S. Figure 4e is the EDX of S3. The results show that the sample contained Zn, O, Cu, and S elements, which further confirmed that the sample may be composed of ZnO NNs and Cu_2_S QDs, and the surface chemical states will be further confirmed by XPS analysis. 

**Figure 5:**
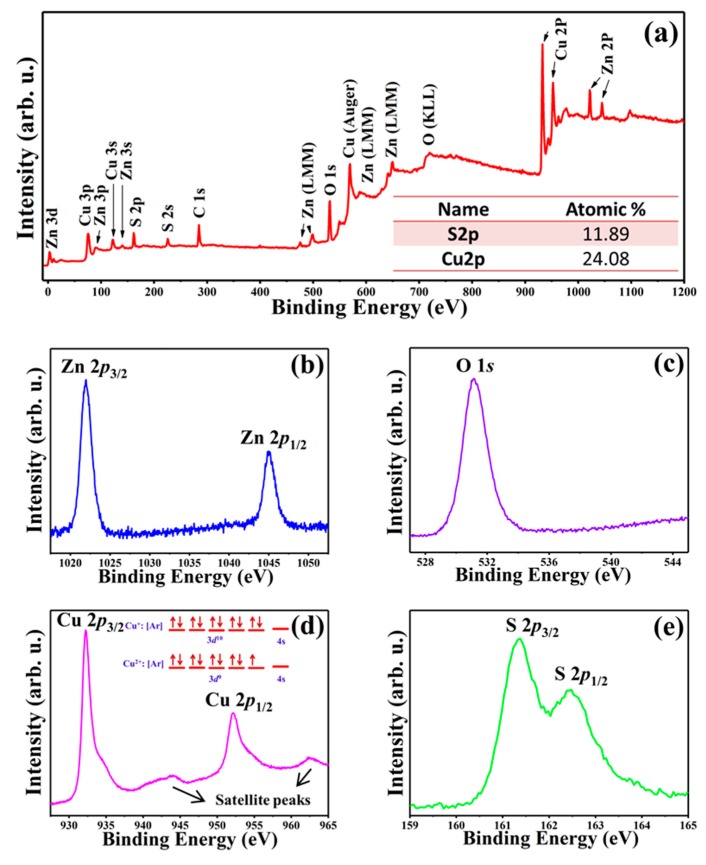
(**a**) XPS spectra of ZnO@Cu_2_S NMSHs (S3, depositing Cu_2_S QDs for six cycles by SILAR method), and High resolution XPS spectra of elemental, (**b**) Zn 2*p*, (**c**) O1*s*, (**d**) Cu 2*p*, and (**e**) S 2*p*.

The XPS measurement was performed to investigate the surface elemental composition and elemental valences of ZnO@Cu_2_S NMSHs (Figure 5a–e). All binding energy values in the XPS spectra were calibrated according to the information of C 1*s* (284.6 eV) [71]. The presence of C element mainly originated from the oil pump owing to vacuum treatment [72]. In the survey spectra of ZnO@Cu_2_S NMSHs (Figure 5a), all elements, namely Cu, Zn, O, and S, are detected with strong characteristic peaks. In addition, the atom ratio of the Cu (24.08, expressing in the illustration of Figure 5a) is two times higher than the S (11.89, expressing in the illustration of Figure 5a) in ZnO@Cu_2_S NMSHs, which has confirmed that the Cu_2_S QDs phase exists in ZnO@Cu_2_S NMSHs. The Zn 2*p* regions of the XPS spectra (Figure 5b) consist of two peaks centered at 1021.9 and 1044.9 eV, which were characteristics of the Zn 2*p*_3/2_ and Zn 2*p*_1/2_ of ZnO [73]. The Zn 2*p* core level dipoles induced by the spin-orbit coupling are typical of ZnO materials in terms of binding energy, peak shape, and peak separation which is 23 eV [74]. The peak centered at 531.2 eV (Figure 5c) corresponds to the O 1*s* peak of ZnO [75]. The Cu 2*p* peaks located at 932.3 eV and 952.2 eV (Figure 5d) are consistent with the binding energies of the Cu 2*p*_3/2_ and Cu 2*p*_1/2_ for Cu^+^ in Cu_2_S, respectively [76]. For the ZnO@Cu_2_S NMSHs, both the asymmetric Cu 2*p* peaks with shoulders on the higher binding energy sides of the Cu 2*p* lines and the satellite peaks which can be found at the higher binding energy direction in Figure 5d can prove that the Cu^2+^ ions are also present in the samples [77,78]. As the illustration of Figure 5d expressed, when the valence of Cu is +1, the outermost electronic configuration of Cu^+^ ions is *d*^10^ arranged with stably and fully paired, so it is difficult to excite the electrons in the d-orbital. However, if the Cu is +2 state, there will be one unpaired electron in the outermost 3*d*-orbital which can easily interact with an out-going electron, absorbed an amount of energy and then jump to a higher energy level. Therefore, if a higher binding energy satellite peaks can be detected by the XPS detector, it is proved that the Cu^2+^ ions exist in the samples [79,80]. Two peaks which are located at 161.3 eV and 162.4 eV (Figure 5e) can be ascribed to S 2*p*_3/2_ and S 2*p*_1/2_ of S^2−^, respectively [81].

### 3.2. Optical Properties

**Figure 6:**
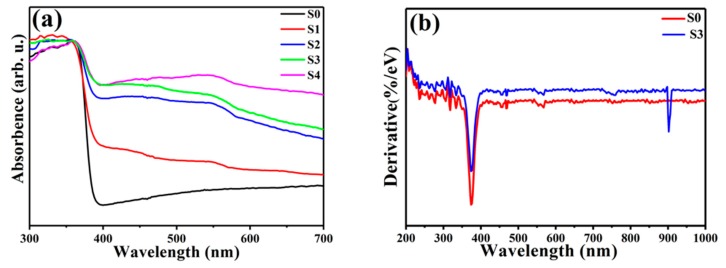
(**a**) UV-Vis spectra of ZnO NNs and ZnO@Cu_2_S NMSHs with depositing Cu_2_S QDs for two cycles (S1), four cycles (S2), six cycles (S3), or eight cycles (S4) by SILAR method and (**b**) the corresponding derivative curves of S0 and S3 samples.

Figure 6a,b shows the room temperature UV-Vis diffuse reflectance spectra in the presence of ZnO NNs (S0) and ZnO@Cu_2_S NMSHs with different SILAR cycle times (S1–S4) and the corresponding derivative curves of ZnO NNs (S0) and ZnO@Cu_2_S NMSHs with six SILAR cycle times (S3), respectively. As Figure 6a shows, the spectrum of pure ZnO NNs (S0) displayed only a sharp UV absorption edge at around 386 nm. The absorption onsets of other samples (S1–S4) are also located at around 386 nm, corresponding to the absorption of ZnO NNs in ZnO@Cu_2_S NMSHs [82]. The almost identical absorption edges of pure ZnO and ZnO@Cu_2_S NMSHs indicate that copper is not doped into the bulk phase of ZnO but exists in the form of sulfide so that composite material is formed with ZnO and Cu_2_S [83]. Compared with the single ZnO NNs, ZnO@Cu_2_S NMSHs also showed an absorption band in the region of 400~700 nm, which should be ascribed to the contribution of Cu_2_S, since its appropriate energy band gap (1.2 eV) structure and the high optical absorption coefficient [84]. It is worth noting that the absorption intensity of this band gradually increased with the increasing amount of Cu_2_S QDs in ZnO@Cu_2_S NMSHs. Figure 6b shows the spectra of S0 and S3 samples deriving from UV–Vis diffuse reflectance spectra (Figure 6a). For S0 sample, only one distinct peak at 374.7 nm (3.31 eV) is observed, which is a characteristic of wurtzite ZnO. For S3 sample, there are two peaks can be observed from the derivative spectrum, which are respectively located at 374.7 nm (3.31 eV) and 902.3 nm (1.37 eV). The peaks at 3.31 and 1.37 eV are ascribed to ZnO NNs and Cu_2_S QDs, respectively. Compared with bulk Cu_2_S material, the band gap of Cu_2_S QDs in ZnO@Cu_2_S NMSHs moves towards higher energy, which may be attributed to quantum size effects [85,86,87].

**Figure 7:**
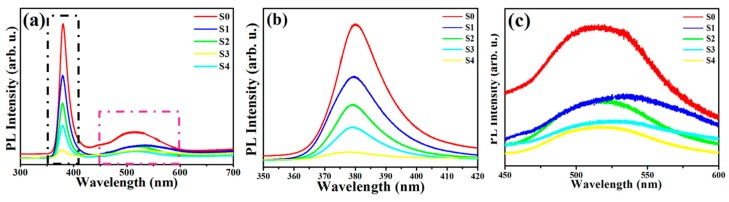
PL (**a**) and magnified PL spectrum (**b**,**c**, the black and pink rectangle in Figure 7a) of ZnO NNs (S0) and ZnO@Cu_2_S NMSHs with depositing Cu_2_S QDs for different cycles by SILAR method (S1, two cycles; S2, four cycles; S3, six cycles; and S4, eight cycles).

Figure 7a–c show the PL spectrum of ZnO NMs and ZnO@Cu_2_S NMSHs at room temperature. It was observed from Figure 7b that the as-prepared ZnO NNs and ZnO@Cu_2_S NMSHs displayed a strong blue emission peak centered at 380 nm, which was close to the near band edge emission of ZnO (ca. 368 nm) due to the recombination of free excitons through an exciton–exciton collision process [88,89,90]. However, besides the fundamental ZnO emission, the green region peaks from 450 nm to 600 nm detected in the PL spectra (Figure 7c) can be generally attributed to oxygen vacancies and surface interstitial oxygen [89,90,91,92], which generally revealed that a number of trapped states have been formed in the forbidden band of ZnO [93]. Such surface defects could act as the recombination centers of photoexcited electron-hole pairs, leading to lower the photocatalytic efficiency. It can be seen from Figure 7a–c, the trend of PL spectrum of ZnO@Cu_2_S NMSHs with different SILAR cycle times is similar to that of single ZnO NNs, but the intensity of those two peaks of ZnO@Cu_2_S NMSHs was lower than the single ZnO NNs, verifying that the ZnO@Cu_2_S NMSHs have higher charge separation efficiency than the pure ZnO NNs. The peak strength has been declining until the SILAR cycle time of Cu_2_S QDs in ZnO@Cu_2_S NMSHs reaches six, which proves that an appropriate increasing amount of Cu_2_S QDs is beneficial to form p–n junctions to promote charge separation. It was reported by Yubin Chen’s group that Cu_2_S could interact with defect states on the surface of CdS and meanwhile quench the emission by forming p–n interfacial junctions. In the p–n nano-match-shaped heterojunctions ZnO@Cu_2_S, similar mechanism can be expected. In other words, it is considered that Cu_2_S QDs can form heterojunctions with ZnO NNs and interact with the surface defects of ZnO (such as oxygen vacancies and surface interstitial oxygen) to quench the surface trap states emission between 450 and 600 nm and increase the charge separation efficiency in ZnO@Cu_2_S NMSHs. When the SILAR reaction cycle reaches eight times (S4), the aggregation of Cu_2_S QDs occurred, and the peak strength of the two PL peaks does not continue to decline but begins to rise because the aggregation of Cu_2_S QDs could restrict the formation of more p–n junctions and the grain boundaries of Cu_2_S QDs would act as the recombination centers, lowing the charge separation [94].

### 3.3. Photocatalytic Performance

**Figure 8:**
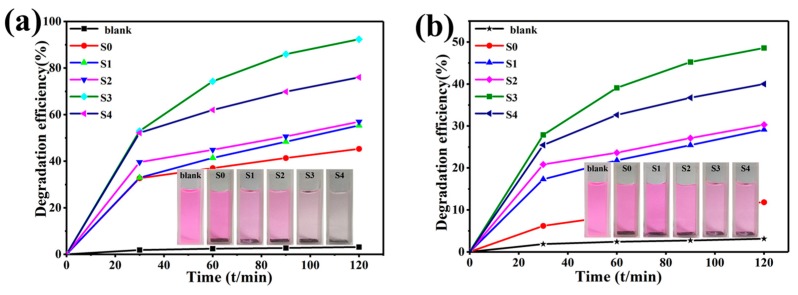
Degradation efficiency as a function of time with ZnO NNs (S0) and ZnO@Cu_2_S NMSHs with depositing Cu_2_S for two cycles (S1), four cycles (S2), six cycles (S3), or eight cycles (S4) under irradiation of (**a**) UV and (**b**) visible light; the color variations of the RhB solution before and after degradation (120 min) for S0–S4 samples as the photocatalysts under the irradiation of UV (the illustrations in (**a**)) and visible (the illustrations in (**b**)).

The photocatalytic performance of the single ZnO NNs and ZnO@Cu_2_S NMSHs with different Cu_2_S QDs deposition amounts has been evaluated toward photocatalytic degradation of RhB under UV and visible light irradiation, respectively. As revealed in Figure 8a,b, the photolysis result of the blank sample which has been performed without photocatalyst is only about 3%, demonstrating that the dye solution is light stable in the absence of photocatalyst. Whether the experiment is done under UV or visible light, the ZnO@Cu_2_S NMSHs have higher photocatalytic degradation efficiency than the ZnO NNs during the entire photocatalytic degradation process (120 min). As the SILAR cycle time of Cu_2_S QDs increases from zero (S0) to two (S1), four (S2), or six (S3), the photocatalytic degradation efficiency performed under UV and visible light has been respectively increasing from 45.3% to 55.3%, 56.9%, and 92.3%, and increasing from 11.8% to 29.1%, 30.3%, and 48.6%, as the SILAR cycle time continues to increase to 8 (S4), the photocatalytic degradation efficiency performed under UV and visible light dose not continue to rise but decreased to 76% and 40%, respectively. Aforementioned results agree well with the color variations of the RhB solution before and after degradation (120 min) for S0–S4 samples as the photocatalysts under the irradiation of UV (the illustrations in Figure 8a) and visible (the illustrations in Figure 8b) light. The enhanced photocatalytic activity was contributed to the combination ZnO NNs with Cu_2_S QDs forming p-n heterojuctions and enhancing specific surface area. When increasing the loading amounts of Cu_2_S on the surface of ZnO NNs, the heterojunctions’ interface of Cu_2_S QDs and ZnO NNs, which can enhance the separation of the generated electron-hole pairs in the presence of light while avoids recombination, will increase at the same time. However, when an excessive amount of Cu_2_S QDs (SILAR cycle for eight times) was used, the more Cu_2_S QDs tended to aggregate together (Figure 3e), which could lead to the increased recombination of photoexcited charges. That is why the photocatalytic degradation efficiency increases first and then decreases. It can be seen that the degradation efficiency of the samples (S0–S4, under UV and visible light) never reached 100 percent, which is mainly ascribed to the existence of CuS phase that the information has been reflected in the XPS results (Figure 5d).

**Figure 9:**
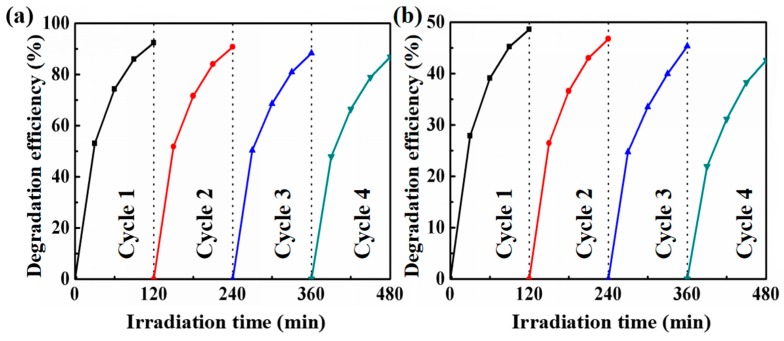
Recycle degradation efficiency of S3 sample (depositing Cu_2_S for six cycles by SILAR method) measured after each interval of 2 h under UV (**a**) and visible (**b**) light.

Photo-stability and reusability is also important for the practical application of photocatalysts. The durability of the ZnO@Cu_2_S NMSHs catalysts for the degradation of RhB under UV and visible illumination was investigated. Following a simple step of washing with water, the recycled photocatalyst was reused and the results of the photocatalyst degradation efficiency of RhB are shown in Figure 9a (UV light) and Figure 9b (visible light). It can be seen from Figure 9a,b that the degradation efficiency of the sample slightly declines after every cycle, probably due to the phase transformation of a little Cu_2_S to CuS [71]. Even so, the photodegradation efficiency of the S3 sample still does not exhibit a significant loss even after the fourth cycle, which indicates that the as-prepared ZnO@Cu_2_S NMSHs catalysts possesses an excellent photostability throughout the photocatalytic process.

### 3.4. Photocatalytic Mechanism

**Figure 10:**
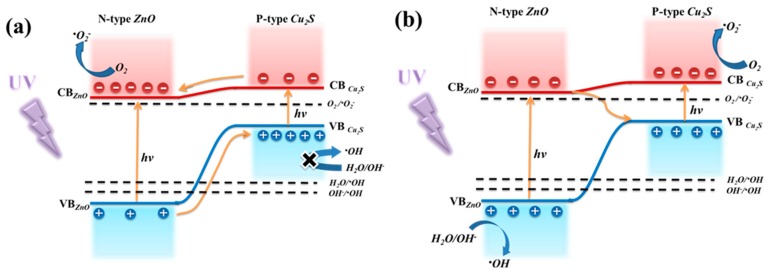
(**a**,**b**) The charge separation process in ZnO@Cu_2_S NMSHs under UV illumination.

According to the PL and UV-Vis results of S0–S4 samples, possible schematic diagram of the charge separation process in ZnO@Cu_2_S NMSHs are illustrated in Figure 10. Where the improved activity of the ZnO@Cu_2_S NMSHs under UV irradiation can be interpreted using two possible routes, as shown in Figure 10a,b. If the electron-hole pairs transfer following the heterojunction mechanism (Figure 10a), both the ZnO NNs and Cu_2_S QDs were excited to generate the electron-hole pairs. The photo-generated electrons are transferred from CB of Cu_2_S QDs into that of ZnO NNs, urging electrons to transfer to O_2_ to yield (superoxide anion radical) •O_2_^−^. At the same time, the photo-induced holes are transferred from valence band (VB) of ZnO NNs into that of Cu_2_S QDs in heterojunction. However, holes were not adsorbed H_2_O or OH^−^ groups into (hydroxyl free radical) •OH, in virtue of the required potential for •OH generation is higher than the VB potential of Cu_2_S QDs [95,96,97,98]. If the photo-generated charges transfer was only carried out according to this process, then the active species that contribute to photocatalytic degradation will mainly consist of (photo-generated holes) h^+^ of Cu_2_S VB and •O_2_^−^, •OH yield by •O_2_^−^ will be just a little amount which can be ignored [99]. Meanwhile the photocatalytic efficiency should be very low, because •OH is well known to be very reactive oxidative species for the oxidation decomposition of organic molecules (RhB) or water pollutants and degrade them, which is inconsistent with the experiment results of Figure 8. Hence, the photo-induced electron-hole transfer in ZnO@Cu_2_S NMSHs may follow basically Z-scheme mechanism [100,101]. As expressed in Figure 10b, since the energy gap between CB of ZnO and VB of Cu_2_S is the smallest, the conduction band electrons of ZnO and the valence band holes of Cu_2_S are easy to recombine. And the photo-generated holes in ZnO remain mainly in its VB to transmit to H_2_O or OH^−^ to form highly reactive •OH, meanwhile the photo-generated electrons in the CB of Cu_2_S are trapped by O_2_ near the surface of Cu_2_S to form reactive •O_2_^−^. Finally, the RhB is degraded by these highly active radical species. The photocatalytic reactions were possibly written as follows [71]:

ZnO/Cu_2_S + *h*γ → Cu_2_S (e + h^+^)/ZnO (e + h^+^) → Cu_2_S (e)/ZnO (h^+^) (1)

e + O_2_ → •O_2_^−^ (2)

h^+^ + OH^−^ → •OH (3)

•O_2_^−^ + H_2_O → •HO_2_ + OH^−^ (4)

•HO_2_ + H_2_O → H_2_O_2_ + •OH (5)

H_2_O_2_ → 2•OH (6)

•OH + RhB → Oxidation products.(7)

It is noteworthy that •OH is mainly derived from the redox reaction of h+, and just a little bit of •OH yield by •O_2_^−^ which can be ignored in this photocatalytic reaction [99].

**Figure 11:**
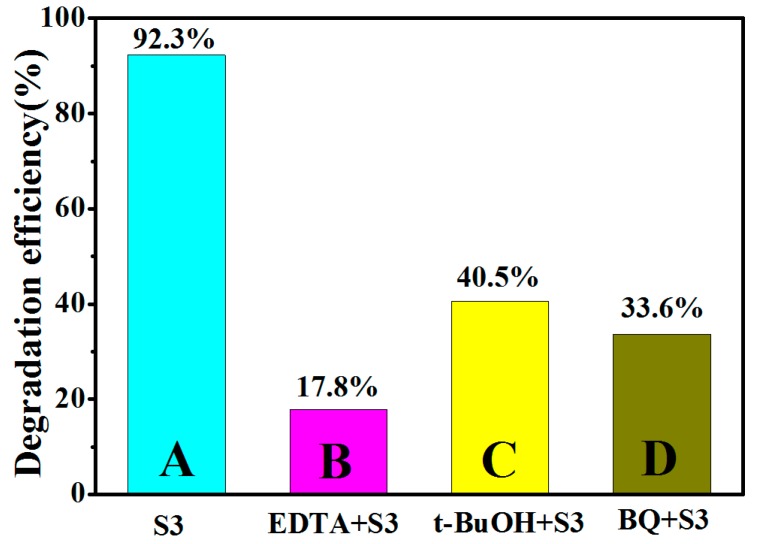
Effect of active species scavengers on percentage photodegradation of RhB using S3 sample (depositing Cu_2_S for six cycles by SILAR method) under UV irradiation.

In order to further prove the basically Z-scheme mechanism under ultraviolet light, the scavenging experiment was performed. The scavenging experiment procedure is similar to the degradation experiment; various scavengers such as ethylene-diamine tetraacetate (EDTA), tert-butyl alcohol (t-BuOH), and 1,4-ben-zoquinone (BQ) were respectively introduced into the aqueous RhB before the addition of photocatalyst to scavenge the h+, •OH and •O_2_^−^ [102,103]. As shown in Figure 11, the histogram from A to D shows the photodegradation efficiency results of scavenging experiment without adding any capture agent, adding h+ trapping agent EDTA, adding an •OH trapping agent t-BuOH, and adding an •O_2_^−^ trapping agent BQ under UV light irradiation. After adding EDTA (capturing the h+), the photodegradation efficiency has dropped from 92.3% to 17.8% caused by the absence of the active species h+. Now the active species which is responsible for photocatalytic degradation efficiency is •O_2_^−^. After adding t-BuOH (capturing the •OH), the active species are •O_2_^−^ and h+, and the photodegradation efficiency is 40.5 % which is much higher than the result of B (17.8 %). After comparing this result with B, it can be inferred that the role of h+ in photocatalytic degradation of RhB is greater than that of •O_2_^−^. Meanwhile, the •OH mainly originated from the oxidation of holes in the valence band of ZnO NNs is generated during catalytic degradation process because the photocatalytic efficiency is reduced after the •OH is captured. Hence, the conclusion that •OH converted from h+ and h+ participate jointly in photocatalytic degradation in this process can be obtained, which is consistent with the basically Z-scheme mechanism in Figure 10b. After adding BQ (capturing the •O_2_^−^), the photodegradation efficiency is 33.6 %, and the active species which is responsible for photocatalytic degradation efficiency are h+ and •OH generated by h+, which proves the existence of h+ and •OH generated by h+ once again, and further proves that the electronic transmission mechanism is the Z-scheme mechanism.

**Figure 12:**
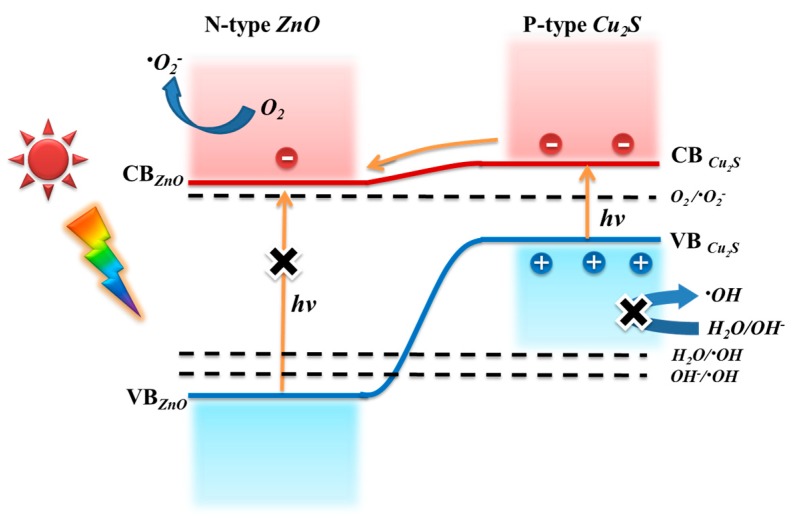
Electron-transfer processes of ZnO@Cu_2_S NMSHs under visible light illumination.

Figure 12 depicts electron-transfer processes of ZnO@Cu_2_S NMSHs under visible light illumination. When visible light is irradiated on ZnO@Cu_2_S NMSHs, only the ground state electronics of Cu_2_S QDs can absorb solar energy and jump to the conduction band, and ZnO NNs can’t, due to its wider band gap (3.37 eV). After exposure to visible light, the holes keep remain in the Cu_2_S QDs valence band and the conduction band (CB) electrons of Cu_2_S QDs transfer to the conduction band of ZnO NNs by electron injection, which helps in the separation of the photo-generated electron-hole pairs and avoids recombination. The electronics in conduction band of ZnO NNs reacted with the dissolved oxygen on ZnO surface to yield the •O_2_^−^ which can continuously participated in the photocatalytic reaction to generate the •HO_2_ and •OH. However, the accumulated photo-generated holes in the valence band (VB) of Cu_2_S QDs can’t react with adsorbed H_2_O or OH^−^ to form •OH radicals, because the valence band potential of Cu_2_S QDs is lower than the •OH. It is possible that the holes themselves directly oxidize the RhB molecules [95,104]. The analysis of above investigation about charge separation process in ZnO@Cu_2_S NMSHs under UV and visible light is consistent with the result of photocatalytic degradation experiments, namely, h^+^ and •O_2_^−^ are main active species under visible light illumination, except for the highly reactive •OH. Therefore, it is easy to understand that the photocatalytic activity under UV light is much higher than the photocatalytic activity under visible light for the same samples.

In the last decade, enormous efforts have been made to prepare different photocatalysts, for example Ag_3_PO_4_/ZnO [105,106], ZnO/CuO [107,108], ZnO@AgI [109,110], TiO_2_/g-C_3_N_4_ [111], MoS_2_@Cu_2_S [112], and so on, for degrading various organic dyes, such as methyl orange (MO) [112,113,114], RhB [105,106,107,108,115], methylene blue (MB) [116,117], phenol [118], etc. It can be seen from Table 1 that even if the components constituting the heterojunction photocatalyst are the same, the photocatalytic degradation efficiency is not the same due to the different conditions in experimental, such as, the amount of catalyst, the type and concentration of organic dyes, and the source used in photocatalytic degradation experiments, the distance between the sample and the source, and the exposure time. The photocatalytic degradation efficiency of the same organic dye is not the same because of the different photocatalysts or experiment conditions used. Thus extensive possibilities exist in this promising area of research, which need to be given full attention and the achievements of such exploration should benefit commercial sector both in terms of ecology and economy.

## 4. Conclusions

In this work, a novel nano-match-shaped ZnO@Cu_2_S photocatalyst with a p-n heterostructure was successfully synthesized. The amount of Cu_2_S QDs in ZnO@Cu_2_S NMSHs, which can be controlled by adjusting the number of SILAR cycles, was the key factor for the photocatalytic performance of the fabricated samples. As the SILAR cycle time of Cu_2_S QDs increases, the photocatalytic performances increases first and then decreases no matter what light sources were used. The enhanced photocatalytic activity was contributed to the combination ZnO NNs with Cu_2_S QDs forming p–n heterojuctions and the declined photocatalytic activity was attributed to the more Cu_2_S QDs tended to aggregate together. It is worth noting that the photocatalytic performance of the same sample irradiated with UV light is much higher than that of visible light. This is because the charge transport of the sample under the illumination of the UV light follows the Z-scheme mechanism, so not only h^+^ and •O_2_^−^ active species but also the highly reactive •OH will be yielded, however, the ZnO in the sample could not be excited to generate electronic-hole pairs while the sample exposed to visible light, so only h^+^ and •O_2_^−^ active species can be produced without the highly reactive •OH. In addition, the photocatalytic efficiency of the S3 sample has no significant decrease even after four cycles, which indicates that the ZnO@Cu_2_S NMSHs photocatalyst exhibits an excellent stability throughout the photocatalytic process. It is believed that the study of the composite materials with p–n heterostructure for high-efficiency photocatalytic applications will contribute to the development of energy conservation and environmental protection.

## Figures and Tables

**Table 1:** List of diverse photocatalysts studied for degrading various organic dyes.

Sample	Amount	Application	Concentration & Usage	Power Source	Time	Efficiency	Ref.
Ag_3_PO_4_/ZnO heterojunction	20 mg	Rh B degradation	10 mg/L 50 mL	Xe lamp 125 mW/cm^2^	15 min	100%	Luo et al. [105]
Ag_3_PO_4_/ZnO heterojunction	50 mg	Rh B degradation	9.0×10^−6^ M, 25 mL	Xe lamp 500 W	40 min	96%	Liu et al. [106]
ZnO/CuO composites	-	MB degradation	10 ppm	halogen lamp 500 W		92.5%	Harish et al. [107]
ZnO/CuO heterostructure	2 × 2 cm^2^	Rh B degradation	1.0 × 10^−5^ M, 40 mL	Mercury lamp 500 W	400 min	100%	Pal et al. [108]
ZnO@AgI hierarchical	50 mg	MO degradation	10 mg·L^−1^ 50 mL	metal halide lamp 70 W	90 min	83.1%	Huang et al. [109]
AgI/ZnO heterojunction	15 mg	Rh B degradation	1.0 × 10^−5^ M, 50 mL	Xe lamp 500 W	150 min	100%	Wang et al. [110]
TiO_2_/g-C_3_N_4_ heterojunction	40 mg	MB degradation	6.0 × 10^−5^ M, 80 mL	LED light 30 W	100 min	100%	Li et al. [111]
MoS_2_@Cu_2_S heterojunction	2.5 mg	MO degradation	6.0 × 10^−5^ M, 20 mL	Xe lamp 300 W	60 min	95%	Zhang et al. [112]
ZnO/Cu_2_S/ZnO complex film	2.5 × 2.5 cm^2^	MO degradation	1.0 × 10^−4^ M, 30 mL	Mercury lamp 175 W	90 min	86%	Wang et al. [113]
ZnO/Cu_2_S/ZnO complex film	2.5 × 2.5 cm^2^	MO degradation	1.0 × 10^−4^ M, 20 mL	Mercury lamp 175 W	90 min	75%	Xu et al. [114]
ZnO/CdS heterojunction	50 mg	Rh B degradation	5.0 × 10^−5^ M, 100 mL	Xe lamp 300 W	90 min	100%	Li et al. [115]
ZnO/SnO_2_ nanocomposites	40 mg	MB degradation	6.0 × 10^−5^ M, 100 mL	Mercury lamp 250 W	80 min	100%	Lin et al. [116]
WO_3_/g-C_3_N_4_ heterojunction	100 mg	MB degradation	3.0 × 10^−5^ M, 100 mL	Xe lamp 300 W	120 min	97%	Huang et al. [117]
Ag_2_CrO_4_-GO composites	20 mg	Phenol degradation	5.0 × 10^−5^ M, 100 mL	Xe lamp 300 W	60 min	90%	Xu et al. [118]
Cu_2_O/ZnO Hetero -nanobrush	-	MO degradation	1.0 × 10^−5^ M	solar simulator 100 mW/cm^2^	120 min	93%	Deo et al. [31]
